# Development of a Flowmeter Using Vibration Interaction between Gauge Plate and External Flow Analyzed by LSTM

**DOI:** 10.3390/s20205922

**Published:** 2020-10-20

**Authors:** Jie Jin, Youngbeen Chung, Junhong Park

**Affiliations:** Department of Mechanical Engineering, Hanyang University, Wangsimni-ro 222, Seongdong-Gu, Seoul 04763, Korea; jinjie@hanyang.ac.kr (J.J.); chung_911@naver.com (Y.C.)

**Keywords:** flowmeter, LSTM, vibration response, mass flow rate, external flow

## Abstract

(1) Background: This study is aimed at the development of a precise and inexpensive device for flow information measurement for external flow. This novel flowmeter uses an LSTM (long short-term memory) neural network algorithm to analyze the vibration responses of the gauge plate. (2) Methods: A signal processing method using an LSTM neural network is proposed for the development of mass flow rate estimation by sensing the vibration responses of a gauge plate. An FFT (fast Fourier transform) and an STFT (short-time Fourier transform) were used to analyze the vibration characteristics of the gauge plate depending on the mass flow rate. For precise measurements, the vibration level and roughness were computed and used as input features. The actual mass flow rate measured by using a weight transducer was employed as the output features for the LSTM prediction model. (3) Results: The estimated flow rate matched the actual measured mass flow rate very closely. The deviations in measurements for the total mass flow were less than 6%. (4) Conclusions: The estimation of the mass flow rate for external flow through the proposed flowmeter by use of vibration responses analyzed by the LSTM neural network was proposed and verified.

## 1. Introduction

For industrial processes, the monitoring of mass flow rate for granular and powdery materials is important for continuous quality monitoring of the external flow [1]. The impact flow meter is widely used for quantification of moving powdery material as external flow measurement by sensing a horizontal impact force produced by falling material [2,3,4]. In addition to industrial processes, the measurements of mass flow rate are important for applications in biomedical engineering fields. Urine flow rate was measured using a conventional weight transducer sensor for the diagnosis and identification of the cause of lower urinary tract symptoms [5]. For internal flows, detection of the flow rate has been proposed by many research groups. Tissue blood flow was evaluated using a laser Doppler flowmeter during vascular responses to physiological stimuli by measuring the index of the average flux of red cells as well as the pulsatile components [6]. A non-contact flow rate sensing method for mixed gas components was proposed based on an optical lens and photo-detector, and the flow rates of each gas component in the gas mixture were identified [7]. An ultrasonic flowmeter was used in natural gas metrology and process control areas based on the principle of the difference in transit time between upstream and downstream flows [8,9]. An electromagnetic flowmeter and a conductance sensor were introduced to measure the gas–oil or gas–water multiphase flow industries [10,11,12]. The accuracy and stability of the above-mentioned flow rate measurement devices were verified. These methodologies were proposed for internal flows. Since these technologies require high accuracy, the sensing equipment applied in the studies was composed of expensive equipment. For application with large quantities in daily life, inexpensive equipment is required.

The impact flow meter has been used commercially to measure the horizontal impact force produced by a falling powdery material. The impact force was measured using a load cell located at the back of the impact plate. With the development of the fourth industrial revolution and deep-learning technology, a flow rate measurement method that combines traditional flow velocity measurement and neural network technologies has been developed. Yazdanshenasshad et al. applied artificial intelligence tools to calibrate an ultrasonic gas flow meter. A multilayer perceptron neural network (MLPNN)-based calibration was proposed for a utility ultrasonic flow meter to induce a better detection of the non-linear effects in the instrument response, such that the prediction error was decreased to 1.21%, which is lower than the 1.47% for the bracketing method. Applying artificial intelligence tools to a flow meter calibration detected non-linear effects in the instrument response, thus reducing errors when compared to traditional method [13]. Kidd et al. proposed a low-error calibration function obtained using machine learning techniques with experimental data for a gas–solid flow meter. The output voltage of a ring-shaped electrode was predominantly a function of the solid mass flow rate and velocity for the flow of bulk solids in a pipeline flow. By incorporating the flow rate in a mathematical model obtained by machine learning, the output voltage was predicted with superior accuracy, enabling accurate mass flow measurement with velocity compensation [14]. Multiphase flow meters (MPFMs) were proposed as diagnostic tools to measure the real-time commingled flow of oil, water, and gas. Barbariol et al. applied unsupervised anomaly detection algorithms for use in an MPFM; thus, this system allows the user to promptly detect anomalous measurements and achieve an indication of the measurement reliability of historical data [15]. Farzaneh-Gord et al. designed and developed an artificial neural network as a fast and accurate method for calculating the thermodynamic properties of natural gas. The ANN system has 23 inputs, including 21 natural gas components, the temperature, and the pressure, as well as two outputs, i.e., the compressibility factor and the speed of sound. In the present study, the speed of sound for natural gas was calculated for the first time. For application to a natural gas flowmeter calibration, the ANN outputs were employed to determine the critical mass flux of the sonic nozzle at different temperatures and pressures [16]. The various deep-learning technologies improve the accuracy of the flow rate measurement and allows improvement in perception capability. LSTM is an artificial recurrent neural network (RNN) architecture with feedback connections used in the field of deep learning. It processes not only a single data set, but also entire sequences. The LSTM model was created to predict the future rate of mutations in a person’s body when affected by COVID-19 [17]. The LSTM network was built to forecast future stock market values for both GOOGL and NKE. The test results confirmed that the model was capable of tracing the evolution of opening prices for both assets [18]. The LSTM method was applied to predict a tourism flow, and experiment results demonstrated that LSTM methods perform better than auto regressive integrated moving average and back-propagation neural networks [19].

This study describes a novel impact flowmeter based on vibration responses from flow–structure interactions with the external flow. The estimation of the flow rate from the vibration responses was performed by LSTM neural network algorithm. The fast Fourier transform (FFT) and short-time Fourier transform (STFT) were applied to analyze the vibration characteristics of the gauge plate depending on the velocity of the external flow. The running water and falling sands were used as the external flow incident on the gauge plate. To estimate the mass flow rate and total mass flow precisely, the vibration level and roughness were used as the input features. The measured flow rate was used for labeling of the output features were employed for model training. The validity of the proposed flowmeter was verified by comparison to actual flow volume.

## 2. Materials and Methods

### 2.1. Principle of Impact Flowmeter with Fluid–Structure Interactions

**Figure 1:**
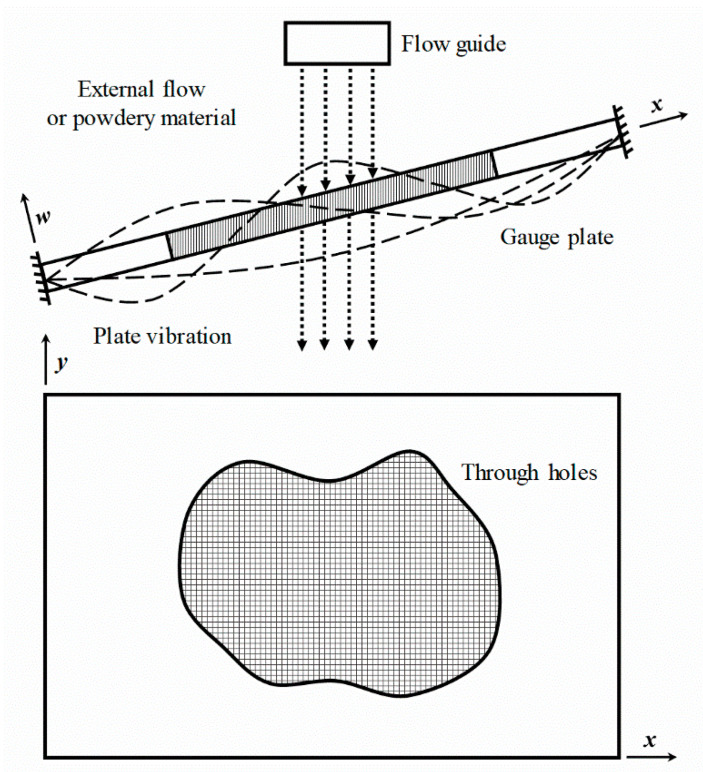
Principle of the flowmeter for measuring the mass flow rate by sensing the vibration characteristics excited by the external flow.

For conventional impact flow meters, the horizontal impact force produced by a falling powdery material is measured by a load cell. In this study, a flow meter using fluid–structure interaction analysis is proposed to determine the mass flow rate and total mass flow of incident external flow or powder materials. To monitor the vibration responses produced by the interaction, the proposed flow meter consists of a frame and a gauge plate. Figure 1 shows a diagram of the gauge plate for analysis of the vibration characteristics excited by the external flow. The center of the gauge plate was designed in the form of a lattice. The panel edges were fixed obliquely on the frame. The lattice structure allowed a sufficient passage of the external flow, thereby preventing the material from embedment to the panel. A flow guide is mounted vertically on the upper surface of the gauge plate grill such that the external flow passes directly through the lattice. The panel vibrates as a result of the fluid–structure interaction due to the moving external flow. A vibration sensor is mounted on the back surface of the gauge plate to measure the resulting vibration responses. By analyzing the vibration characteristics, the real-time mass flow rate can be measured using the LSTM neural network algorithm.

### 2.2. Process of LSTM for Mass Flow Rate Measurements

**Figure 2:**
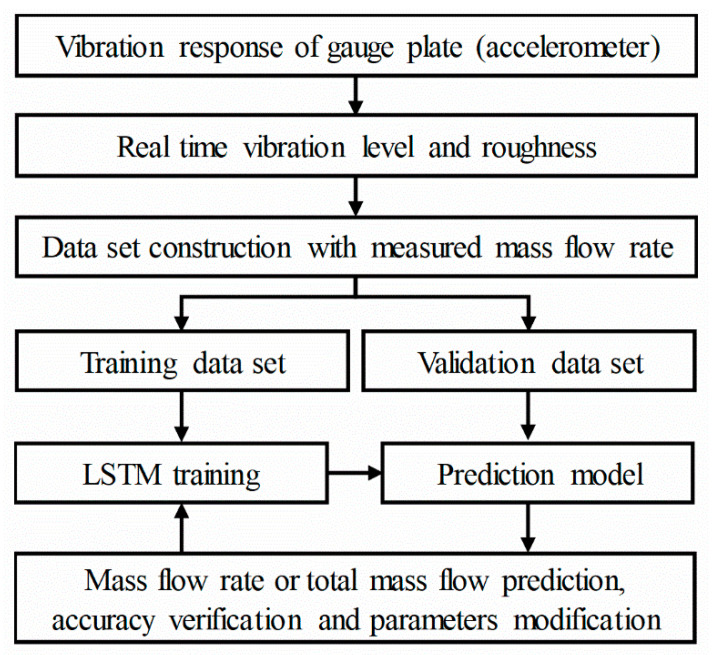
Framework of the proposed LSTM neural network for predicting the mass flow rate based on the vibration signals.

LSTM was used for estimation of the mass flow rate from the vibration responses induced by fluid–structure interactions. LSTM is based on an upgraded RNN architecture that supplements the shortcoming of the reduced learning ability owing to a decrease in the back-propagation slope caused by an increase in the length of the RNN. The LSTM network adds a function for preserving older memory while deleting memory that is no longer needed, which makes it suitable for the classification, processing, and prediction of time-series data. Figure 2 shows the overall flowchart of the present study. An experiment was conducted to measure the vibration of the gauge plate. To enhance the accuracy of the LSTM predictions, a dataset was constructed using the vibration level (the magnitude of the vibration signal measured by the accelerometer) and roughness (pattern of the signal change) as the input features and the flow rate measured by the weight sensor as the output feature. A portion of the dataset was used for training the LSTM deep-learning algorithm for the prediction of the mass flow rate sequence, while the remaining dataset was used to confirm the accuracy of the training model and for an optimization of the deep-learning parameters. Based on the constructed prediction model, the mass flow rate and total mass flow were measured from the vibration of the gauge plate.

### 2.3. Experimental Setup for Gauge Plate Vibration and Mass Flow Rate Data Collection

**Figure 3:**
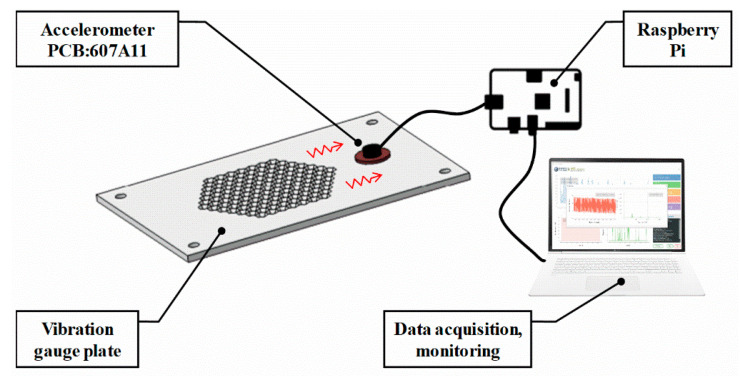
Impact flowmeter system based on the LSTM deep-learning algorithm when using a vibration accelerometer.

Figure 3 shows a diagram of the mass flow meter system used for data collection with a vibration accelerometer. A gauge plate was fabricated through additive manufacturing using a 3D printer. The dimensions were 275 × 150 × 5 mm. The fixed–fixed boundary condition was constructed by mounting the gauge plate to the frame using bolts. To reflect the vibration information of the gauge plate according to the flow, a 13-mm-diameter hole was manufactured 20 mm away from the lattice through hole of the gauge plate for accelerometer fixing. For accurate sensing, an accelerometer with high sensitivity (PCB, 607A11, 10.2 mV/(m/s^2^)) was installed at the fixing hole, and the sampling frequency was set to 10 kHz for response detecting every 0.1 ms. The Raspberry Pi plugs into a computer to form a data collection and monitoring device.

**Figure 4:**
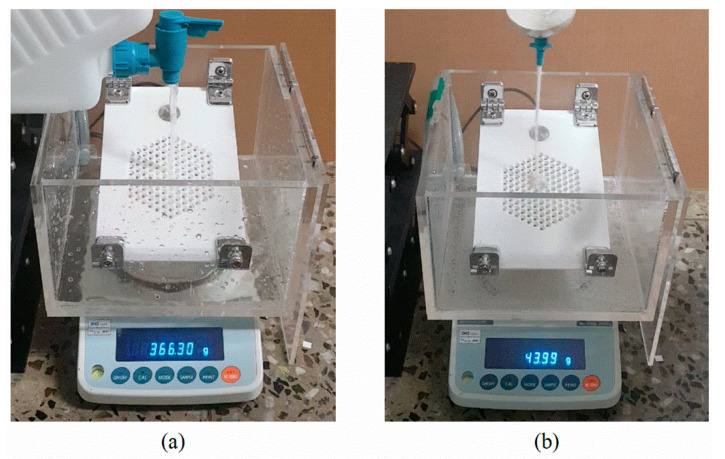
Experimental setup for measuring the vibration of a gauge plate and the mass flow rate of (**a**) running water and (**b**) sands, respectively.

Figure 4 shows the experimental setup for measuring the mass flow rate from the vibration of the gauge plate and the mass flow rate with weight scale data set construction. The gauge plate was attached to a sealed frame to accommodate flowing material, and was set on a weight scale (AND, FX5000i). A rectangular distilled water bottle (Jeio tech, Daejeon, South Korea, DBC-05) filled with water was fixed to a specially designed jig which can maintain a constant distance of 130 mm between the lattice of the gauge plate and the mouthpiece cock. By adjusting the opening angle of the mouthpiece cock, the stability of the reference flow was secured, and the flow rate of the voiding material was adjustable. The real-time weight data of the discharged flowing material and the final mass were collected every 0.2 s with the weight scale connected to the computer. Thus, a database for deep-learning algorithm training was constructed using the vibration signals measured by the accelerometer.

**Figure 5:**
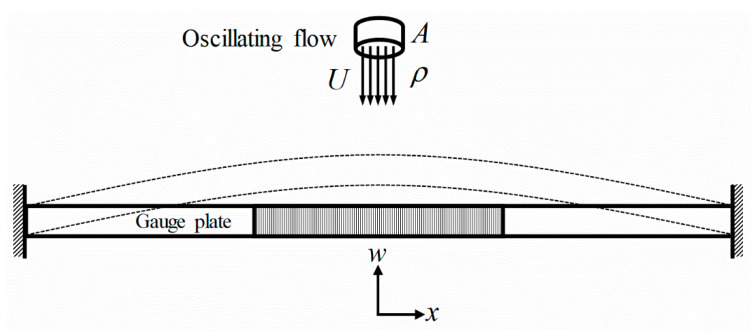
Diagram of excitation force modeling of flowing medium for analyzing vibration interaction between moving medium and the gauge plate restrained at both ends.

To predict the characteristics of the vibrational mechanism of the gauge plate owing to the interaction effect of the moving medium, the wave propagation method was applied. Figure 5 shows the diagram of excitation force modeling of flowing medium for analyzing vibration interaction effect between the gauge plate and moving medium. The gauge plate was assumed to be a classical beam, and the equation for the motion for bending vibration was given as

(1) $$EI\frac{\partial^{4}w\left(x,t\right)}{\partial x^{4}}+M_{b}\frac{\partial^{2}w\left(x,t\right)}{\partial t^{2}}=F_{0}\left(t\right),$$

where *E*, *I*, and *M_b_* are the Young’s modulus, area moment of inertia, and mass per unit length of the beam, respectively. Since the response at the center of the gauge plate is directly affected by the oscillating flow force (*F*_0_) of the moving medium, oscillating force should be derived for determination of the vibrational responses in the frequency range and prediction of the characteristics of the vibrational mechanism of gauge plate interacting with moving medium. The oscillating force due to external flow medium was simulated using the Morison approach, which is the sum of an inertial force and drag force [20]. The inertial force is due to fluid acceleration and the drag force is associated with relative velocity. Owing to the gauge plate being restrained at both ends, the drag force effect was small enough to ignore, and only the inertia force was considered in this study. Thus, the equation of force was estimated as

(2) $$F_{0}\left(t\right)=\rho A\dot{U}\left(t\right)+C_{a}\rho A\left[\dot{U}\left(t\right)-\ddot{w}\left(t\right)\right],$$

which is the sum of the buoyancy and added mass components. Herein, *ρ*, *A*, and *U*(*t*) are the density of the moving medium, the cross-sectional area, and flowing velocity, respectively. *C_a_* and *w*(*t*) are the added mass coefficient and displacement of the gauge plate center position, respectively. The vibration responses of the beam were represented by using spectral representation, the fixed–fixed boundary condition and the continuous condition were applied [21]. Thus, the response of the gauge plate directly affected by the oscillating force owing to the moving medium was measured. The frequency components were obtained by the Fourier analysis as follows:

(3) $$F\left(k\right)=\sum_{n=0}^{N-1}a\left(n\right)e^{-j\left(2\pi k/N\right)n},$$

where *a* is the discrete acceleration response to be transformed, and *N* is the number of periodic finite time and frequency samples. To examine the variation in the spectral vibration response of the gauge plate depending on different flow rates, STFT was performed on the discrete acceleration response as:

(4) $$X\left(k,m\right)=\sum_{n=-\infty }^{\infty }a\left(n\right)W\left(n-m\right)e^{-j\left(2\pi k/N\right)n},$$

where *W* is the window function, and *m* is the shifting amount of window function. To reduce leakage during Fourier transforms, the Hanning window was applied to the time domain data.

### 2.4. Calculation of the Vibration Level and Roughness of the Gauge Plate

As input parameters to the LSTM prediction model, the vibration level, representing the magnitude of the vibration, and roughness, indicating the frequency distribution characteristics, were used. The vibration level was obtained by converting the amount of vibration into a decibel level as follows:

(5) $$L={20\log}_{10}\left[a_{\mathrm{rms}}\left(t\right)\right]-{20\log}_{10}\left[a_{\mathrm{ref}}\right],$$

where *a*_rms_ is the root mean square value of the instantaneous values for a certain time duration, and *a*_ref_ = 10^−5^ m/s^2^ is the reference acceleration level. When the vibration response is modulated at a frequency between 20 and 300 Hz, a change in vibration response according to the change in time cannot be felt, and only the roughness of the vibration response is significant. This achieves the highest sensitivity at a modulation frequency of 70 Hz, and can be expressed as follows:

(6) $$R=0.3\frac{f_{\mathrm{mod}}}{\mathrm{kHz}}\int_{0}^{20Bark}\frac{\Delta L\left(z\right)\mathrm{dz}}{dB/Bark},$$

where *f*_mod_ and Δ*L* represent the modulation frequency and masking depth, respectively. In the present study, the dataset was constructed using the vibration level and roughness for responses of the gauge plate. The mass flow rate was obtained by the weight balance. whereas a flow rate prediction model with robustness and high accuracy in the surrounding environment was constructed based on LSTM neural network algorithm training.

### 2.5. LSTM Mass Flow Rate Prediction Model

**Figure 6:**
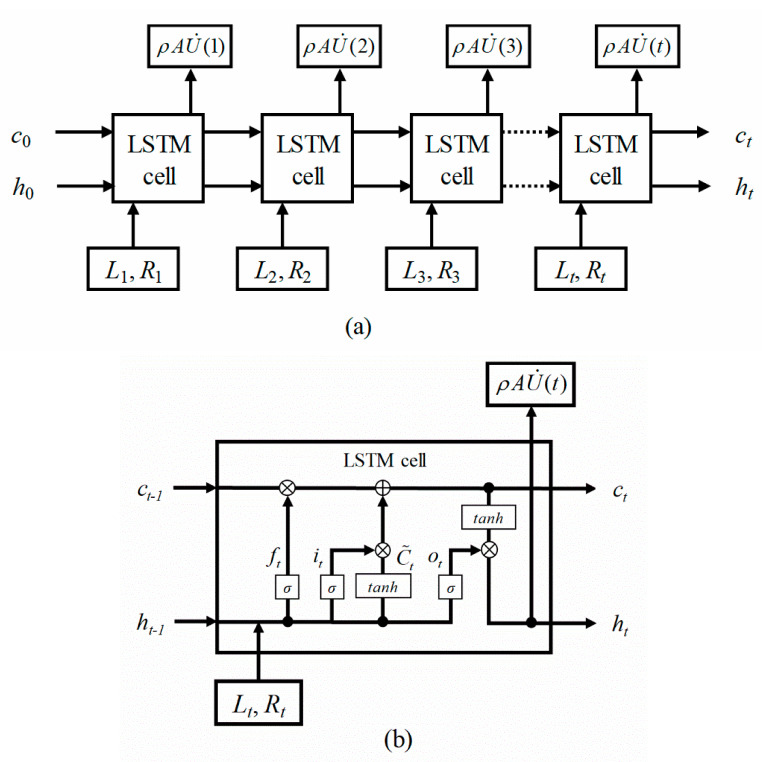
(**a**) LSTM neural network used to predict the transient flow rates with the vibration responses (level and roughness) as the input features, and the measured flow rate as the output features. (**b**) Diagram structure of LSTM cell state.

LSTM can process data, regardless of how long the sequence is, without the problem of a disappearing slope. Figure 6 shows an LSTM network for determining the mass flow rate. This LSTM network consists of multiple cells. Each unit cell has an input gate, an output gate, a forget gate, and a memory cell state. The cells remember the values over an arbitrary time interval, whereas the three gates control the flow of information into and out of the cells. When the input vector constructed with the vibration level and roughness [*x_t_* = (*L_t_*, *R_t_*)] and the previous output values (*h_t_*_−1_) are given, the first step in the forget gate layer of LSTM cell is to determine which information is to be thrown away, which is carried out as follows

(7) $$f_{t}=\sigma \left(W_{f}\left[h_{t-1},x_{t}\right]+b_{f}\right),$$

The next step is to decide what information is to be saved in the input gate layer. A vector of new candidate values is added to the state as follows

(8) $$\begin{array}{c} i_{t}=\sigma \left(W_{i}\left[h_{t-1},x_{t}\right]+b_{i}\right)\\ \tilde{C_{t}}=\tan h\left(W_{c}\left[h_{t-1},x_{t}\right]+b_{c}\right) \end{array},$$

The output value from the forget gate (*f_t_*) is multiplied to forget a certain amount of the cell state values (*c_t_*_−1_), whereas the input (*x_t_*) and previous output values (*h_t_*_−1_) are multiplied by the processed output values of the input gate (*i_t_*) to accept a certain amount as input values, by which a new cell state (*c_t_*) is created as follows

(9) $$c_{t}=f_{t}c_{t-1}+i_{t}\tilde{C_{t}},$$

Finally, the output value (*o_t_*) under this cell state is multiplied as the output of the LSTM cell (*h_t_*). This determines how much to forget from the cell state value, and how much of the input value should be newly accepted.

(10) $$\begin{array}{c} o_{t}=\sigma \left(W_{o}\left[h_{t-1},x_{t}\right]+b_{o}\right)\\ h_{t}=o_{t}\tan h\left(c_{t}\right) \end{array},$$

where *W*, *σ*, tanh, and *b* represent the linear transformation matrix, sigmoid function, hyperbolic tangent, and bias term, respectively [22]. The present study proposes an LSTM for the real-time flow rate estimation of the external flow and carries out a training of the LSTM model with the level and roughness of the gauge plate vibrations as the inputs (*L_t_*, *R_t_*), and the mass flow rate measured by the weight balance as the output [*h*(*t*) = *ρAU*(*t*)].

### 2.6. Verification of the Accuracy of LSTM Prediction Model

To assess the performance of the LSTM model, actual values were plotted. The uncertainty analysis was performed for verification of the measurement performance. For the evaluation of uncertainty, the standard type-A uncertainty calculation was used [23]. The uncertainty was evaluated as

(11) $$u_{A}\left(q\right)=\sqrt{\frac{1}{N\left(N-1\right)}\sum_{i=1}^{N}{\left[q\left(i\right)-Q_{m}\right]}^{2}},$$

where *Q_m_* is the average value of mass flow rate. For the evaluation of the differences between values estimated by LSTM and those measured by the weight balance, the root mean square error of the flow rate was calculated as

(12) $$\gamma \;={\left\{\sum_{i=1}^{\;N}{\left[q_{m}\left(i\right)-q_{p}\left(i\right)\right]}^{2}/N\right\}}^{1/2},$$

where *q_m_* and *q_p_* are the measured and estimated mass flow rates, respectively. The relative error was evaluated as

(13) $$\delta =\frac{\left|M_{m}-M_{p}\right|}{M_{m}}\times 100\%,$$

where *M_m_* and *M_p_* are the measured and estimated total flow mass, respectively.

## 3. Results

### 3.1. Analysis of Vibration Characteristics of the Gauge Plate

**Figure 7:**
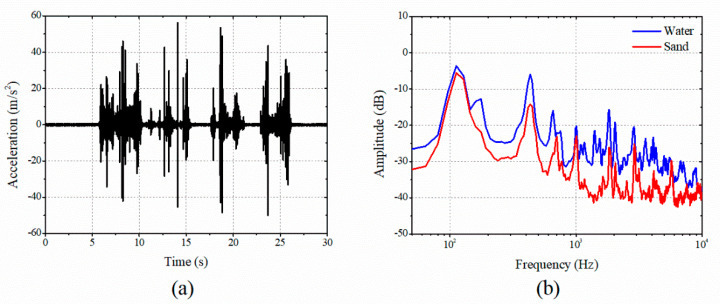
Signal processing of vibrational response of gauge plate: (**a**) time response excited by water and (**b**) frequency responses.

Figure 7 shows the measured gauge plate vibrations excited by the water flows. The rigid body translational resonance of the gauge plate occurred at 112 Hz. The clamped ends effectively prevented rotational movements. The fixture at the plate edges supported the plate with clamped boundary conditions. The plate with holes exhibited much smaller stiffness compared to the fixture. Consequently, the gauge plate vibrations were contributed only by the plate modal properties and were measured using the accelerometer as shown in Figure 7b. Experiments were performed using the sands and water as the external flow medium. Notes that the sand is a powder material and possesses the characteristics of a fluid when flowing without constraints from boundaries [24]. The response of the gauge plate is affected by the medium speed and modal characteristics of the gauge plate. The FFT technique was applied by capturing the vibrational response at a constant mass flow rate measured by the scale. The vibration response of the gauge plate due to the high mass flow rate of water omission was larger than that of sand, resulting in more considerable amplitude. Since the boundary condition and excitation location of the gauge plate are constant, there is no difference in the mode frequency band, but there is a difference in the magnitude of the vibration response. To obtain the natural frequency, the gauge plate was analyzed as a Euler–Bernoulli beam. The deflection was calculated with continuous vibrating system. For a clamped beam fixed at both ends, the natural frequency is given as *ω_n_* = (*β_n_l*)^2^(*EI*/*ρAl*^4^)^1/2^ [25]. In Table 1, the measured and predicted natural frequencies are compared. When the mechanical properties of acrylonitrile butadiene styrene in the gauge plate were *E* = 1.4 GPa, *I* = 1.5625 × 10^−9^ m^4^, *ρ* = 1015 kg/m^3^, *A* = 7.5 × 10^−4^ m^2^, and *l* = 0.275 m, the predicted natural frequencies were in good agreement with the measured results.

**Figure 8:**
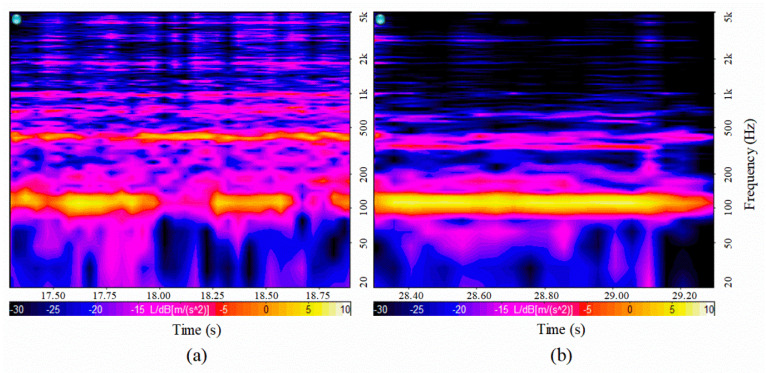
Comparison of gauge plate vibration using an STFT depending on the different flow velocities: (**a**) 1.35 and (**b**) 1.03 g/s.

Figure 8 compares the STFT of the vibration responses when the flow velocities were 1.35 and 1.03 g/s, respectively. As the velocity of the external flow increases, the magnitude of the excitation force increases accordingly. Thus, an increase in the vibration response appears throughout the entire frequency range. As the mass flow rate increased, the vibration responses at the high frequency modes of the gauge plate increased significantly. The level represented the magnitude of vibration. The roughness indicated the spectral characteristics of the vibration. These two parameters were used as the input features for the training of the LSTM algorithm.

### 3.2. Comparison of Vibration Responses and Mass Flow Rate

**Figure 9:**
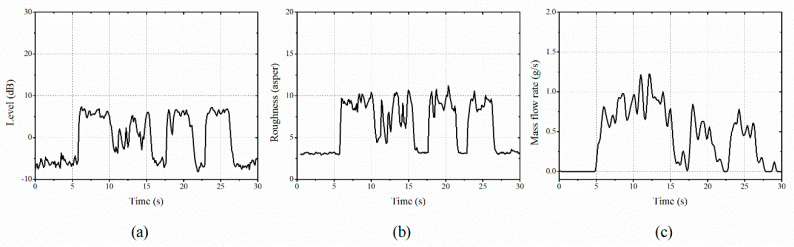
Comparison of the vibration signals and mass flow rate: (**a**) vibration level, (**b**) roughness, and (**c**) mass flow rate.

Figure 9 shows a comparison between the measured real-time vibration level and roughness and its comparison with the mass flow rate obtained by the weight sensor. The stem size of the flow was related to the excitation force amplitude of the gauge plate. The flow velocity was related to the excitation frequency of the gauge plate. This affects the spectral characteristics of the vibration responses. The pattern extracted by the calculation of two real-time vibration factors of the gauge plate showed a similar variation to that of the mass flow rate. In the present study, 190 datasets were constructed using the measured vibration level and roughness of the gauge plate as input features, and the measured flow rate as the output for the neural network training.

### 3.3. Performance Validation of the Proposed Flow Meter Based on Neural Network

**Figure 10:**
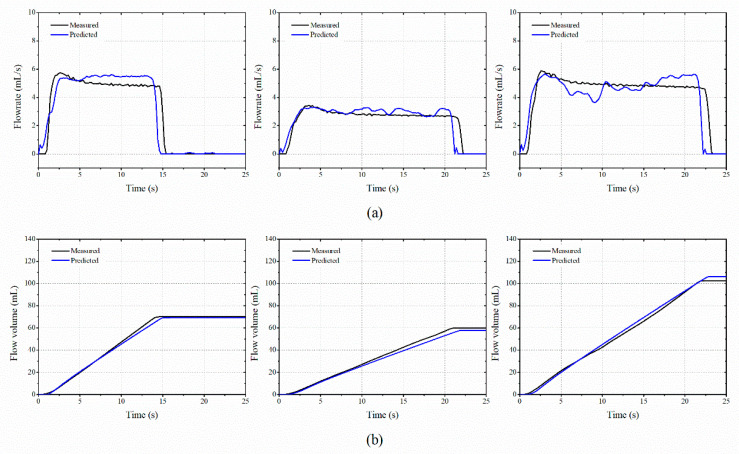
Comparison of the measured water flow rate by using the proposed LSTM and vibration responses: (**a**) mass flow rate and (**b**) total mass flow.

**Figure 11:**
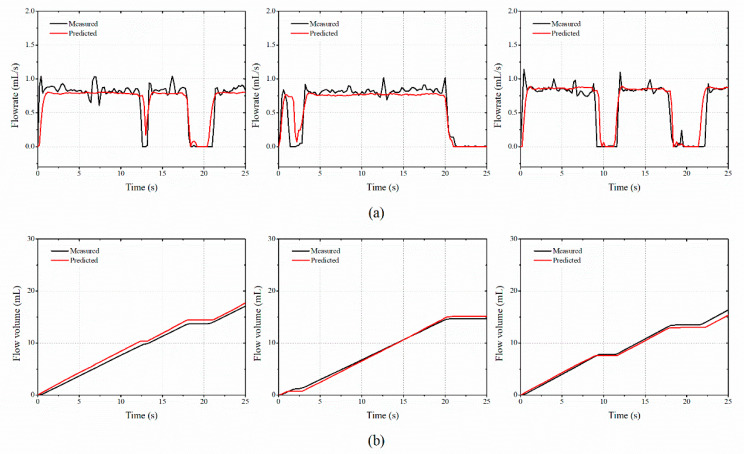
Comparison of the measured sand flow rate by using the proposed LSTM and vibration responses: (**a**) mass flow rate and (**b**) total mass flow.

When creating a deep-learning-based model, the volume of the learned data, the attributes, and the variable settings were important. The look back, hidden unit, batch size, and epoch were set to 5, 64, 1, and 300, respectively. For the learned state from one batch was carried over to learn the subsequent batch, the optimizer and stateful options of the LSTM model were set as ADAM and true, respectively. A dense layer with only one neuron was used to carry out the training by the LSTM model. Figure 10 shows the predicted mass flow rate and total mass of the water by using the proposed LSTM algorithm. The *γ* values for the mass flow rate were 0.9837, 0.4129, and 0.7699 for separate tests. The *δ* values for the total mass of the arbitrary samples were 1.26%, 3.38%, and 3.83% for separate tests. Figure 11 represents the results for the sand medium. The *γ* values were 0.1773, 0.1506, 0.1960, and the *δ* values were 2.65%, 3.16%, 4.84% for separate tests. Table 2 shows the *γ* and *δ* values for the tested cases. For the sand medium with a relatively high density, it stably passed through the lattice of the gauge plate. When the water passed through the lattice, it bounced in all directions. The fine cavitation effect also influenced the measurement deviations. Thus, the *γ* of the mass flow rate of the water medium was four times larger than that of the sand. When compared to the difference in *γ*, the deviations of *δ* for total mass between water and sand medium become small.

**Figure 12:**
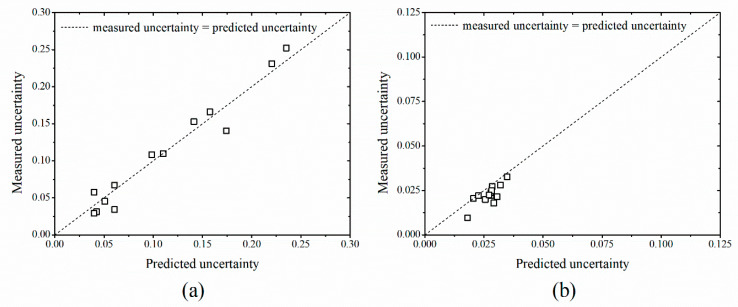
Comparison of the uncertainty of estimated and measured mass flow rate for (**a**) water and (**b**) sand.

The type-A uncertainty coefficients extracted from the estimated real-time mass flow rate are shown in Figure 12. Compared with the water for which the rates easily by the turbulent flows, the uncertainty value calculated for the sand was small. The standard deviation for the water was 0.0178, which is larger than the value of 0.00637 calculated for the sand. The uncertainty of the mass flow rate estimated by LSTM model showed a similar level both for water and sand. This confirmed the validity of the proposed methodology. This confirmed the potential for the development and realization of an LSTM deep-learning-based flow rate measurement that is robust against influence from surrounding environments.

## 4. Conclusions

In the present study, an LSTM flow rate detection methodology using the vibration responses of a gauge plate was proposed. The spectral characteristics were analyzed to investigate the variation of the gauge plate vibration depending on the mass flow rate. As the velocity or density of the external flow increases, the vibration response over the entire frequency domain and the high-frequency mode responses significantly increased. LSTM was advantageous for improving the estimation accuracy when various parameters were applied as inputs, rather than a single parameter. For more accurate estimations, the transient variations of vibration level and roughness were used as the input features. The mass flow rate measured using a weight transducer was applied as output to construct the LSTM training dataset. The training of a deep-learning algorithm was carried out to build the LSTM flow rate estimation. The estimated and actual measured flow information were very similar. The LSTM model showed good performance both in water and sand medium flux. The continuous monitoring of the mass flow rate and total mass flow through the vibration-based LSTM model was confirmed via experimentation. This algorithm is applicable in portable devices in different environments including flow rate measurements for IoT.

## 5. Patents

A patent (P20190907OP) titled “Development of healthcare urinary flow rate measurement device using LSTM deep-learning prediction” was filed based on the technology developed in this study. This technology is an LSTM algorithm for predicting a urinary flow rate index related to the lower urinary tract symptoms required in the flowmetry test based on the vibroacoustic signals.

## Figures and Tables

**Table 1:** Comparison of the measured and predicted natural frequencies.

Mode	1st	2nd	3rd	4th	5th	6th	7th	8th
*β_n_l*	3.9	7.1	10.2	13.3	16.5	(4*n*+1)π/4*, n > 5*		
Experiment (Hz)	177	420	655	1004	1408	1815	2044	2875
Predict (Hz)	178	372	636	971	1375	1851	2397	3013

**Table 2:** Comparison of the root means square error of mass flow rate and relative error of the total mass.

Water	*u_A_*		*γ*	*δ* (%)	Sand	*u_A_*		*γ*	*δ* (%)
	Measure	Predict				Measure	Predict		
1	0.0280	0.0220	0.9837	1.2565	1	0.2205	0.2310	0.1773	2.6460
2	0.0320	0.0280	0.4129	3.3772	2	0.0985	0.1083	0.1503	3.1626
3	0.0306	0.0214	0.7699	3.8343	3	0.1576	0.1660	0.1960	4.8402
4	0.0205	0.0205	0.7032	4.5905	4	0.1414	0.1528	0.1556	5.3024
5	0.0279	0.0237	1.1567	11.0908	5	0.2353	0.2521	0.1597	6.9358
6	0.0285	0.0272	0.922	0.2108	6	0.1744	0.1406	0.1396	5.5649
7	0.0348	0.0326	0.4696	3.6737	7	0.0400	0.0578	0.1738	7.6984
8	0.0282	0.0248	0.4878	10.0374	8	0.0608	0.0670	0.103	3.7704
9	0.0292	0.0179	0.3586	1.5855	9	0.0426	0.0313	0.1292	5.9304
10	0.0181	0.0096	0.2793	7.6147	10	0.0608	0.0342	0.1007	0.9887
11	0.0227	0.0221	0.3562	8.7841	11	0.0398	0.0292	0.1607	7.0165
12	0.0256	0.0199	0.3476	8.6268	12	0.0508	0.0453	0.1905	8.4739
Average	0.0272	0.0225	0.6040	5.3902	Average	0.1102	0.1096	0.1533	5.3739

## References

[1] Harris B., Davies C., Davidson J. (1997). The slot flow meter: A new device for continuous solids flow measurement. Chem. Eng. Sci..

[2] Tomiyasu H., Tanaka H. (1984). Impact Flow Meter. U.S. Patent.

[3] Kajiura H., Watanabe K. (1971). Impact Flow Meter for Powdery and Granular Materials. U.S. Patent.

[4] Kempf D., McCarthy W.P. (1994). Impact Flowmeter. U.S. Patent.

[5] Nordling J. (2002). The aging bladder—A significant but underestimated role in the development of lower urinary tract symptoms. Exp. Gerontol..

[6] Nilsson G.E., Tenland T., Oberg P.A. (1980). Evaluation of a laser Doppler flowmeter for measurement of tissue blood flow. IEEE Trans. Biomed. Eng..

[7] Han B., Zhang Y.-N., Wang X., Zhou F.-D., Li T., Guo H., Chen S.-S., Yuan J.-L. (2018). Non-contact flow rate detection of component in mixed gas using spectrum absorption theory. Opt. Fiber Technol..

[8] Zhou H., Ji T., Wang R., Ge X., Tang X., Tang S. (2018). Multipath ultrasonic gas flow-meter based on multiple reference waves. Ultrasonics.

[9] Sun Y., Zhang T., Zheng D. (2018). New analysis scheme of flow-acoustic coupling for gas ultrasonic flowmeter with vortex near the transducer. Sensors.

[10] Yang Q.-Y., Jin N.-D., Zhai L.-S., Ren Y.-Y., Yu C., Wei J.-D. (2020). Measurement of Water Velocity in Gas–Water Two-Phase Flow with the Combination of Electromagnetic Flowmeter and Conductance Sensor. Sensors.

[11] Meribout M., Azzi A., Ghendour N., Kharoua N., Khezzar L., AlHosani E. (2020). Multiphase Flow Meters Targeting Oil & Gas Industries. Measurement.

[12] Wang Y., Li H., Liu X., Zhang Y., Xie R., Huang C., Hu J., Deng G. (2016). Novel downhole electromagnetic flowmeter for oil-water two-phase flow in high-water-cut oil-producing wells. Sensors.

[13] Yazdanshenasshad B., Safizadeh M. (2018). Neural-network-based error reduction in calibrating utility ultrasonic flow meters. Flow Meas. Instrum..

[14] Kidd A.J., Zhang J., Cheng R. (2020). A low-error calibration function for an electrostatic gas-solid flow meter obtained via machine learning techniques with experimental data. Energy Built Environ..

[15] Barbariol T., Feltresi E., Susto G.A. (2019). Machine Learning approaches for Anomaly Detection in Multiphase Flow Meters. IFAC PapersOnLine.

[16] Farzaneh-Gord M., Mohseni-Gharyehsafa B., Ebrahimi-Moghadam A., Jabari-Moghadam A., Toikka A., Zvereva I. (2018). Precise calculation of natural gas sound speed using neural networks: An application in flow meter calibration. Flow Meas. Instrum..

[17] Pathan R.K., Biswas M., Khandaker M.U. (2020). Time Series Prediction of COVID-19 by Mutation Rate Analysis using Recurrent Neural Network-based LSTM Model. Chaos Solitons Fractals.

[18] Moghar A., Hamiche M. (2020). Stock Market Prediction Using LSTM Recurrent Neural Network. Procedia Comput. Sci..

[19] Li Y., Cao H. (2018). Prediction for tourism flow based on LSTM neural network. Procedia Comput. Sci..

[20] Blevins R.D. (1977). Flow-Induced Vibration.

[21] Ahn S., Koh H., Lee J., Park J. (2019). Dependence between the vibration characteristics of the proton exchange membrane fuel cell and the stack structural feature. Environ. Res..

[22] Sherstinsky A. (2020). Fundamentals of recurrent neural network (rnn) and long short-term memory (lstm) network. Phys. D.

[23] Bich W., Cox M.G., Harris P.M. (2006). Evolution of the ‘Guide to the Expression of Uncertainty in Measurement’. Metrologia.

[24] Savage S.B., Hutter K. (1991). The dynamics of avalanches of granular materials from initiation to runout. Part I: Analysis. Acta Mech..

[25] Inman D.J., Singh R.C. (1994). Engineering Vibration.

